# The genetic pathways involved in sensory hypersensitivity and related psychiatric disorders

**DOI:** 10.1192/j.eurpsy.2026.11273

**Published:** 2026-07-31

**Authors:** N. Bouayed Abdelmoula, B. Abdelmoula, S. Kotti, A. Etteyeb

**Affiliations:** LR23ES07 Genomics of Signalopathies at the service of Precision Medicine, Medical University of Sfax, Sfax, Tunisia

## Abstract

**Introduction:**

Sensory hypersensitivity, characterized by increased responses to sensory stimuli, is a common feature of several psychiatric and neurodevelopmental disorders. Genetic factors influencing neurotransmission and the function of neural circuits are increasingly recognized as key contributors to this complex phenotype.

**Objectives:**

This review synthesizes recent findings on the genetic pathways involved in sensory hypersensitivity, focusing on neurotransmitter systems, synaptic plasticity and neuro-immune interactions identified through genomic and neurobiological studies.

**Methods:**

We conducted a comprehensive review of the scientific literature using as key words: sensory hypersensitivity, genetics, signaling pathways, psychiatry.

**Results:**

Sensory hypersensitivity and associated disorders, often called sensory processing disorder (SPD), involves an atypical increased sensitivity and an excessive reaction to sensory stimuli such as images, sounds, touch, smells, tastes, movements or internal bodily sensations. People with sensory hypersensitivity may find the daily sensory input overwhelming or even painful, resulting in strong emotional and behavioral responses. This phenomenon is commonly observed in a range of psychiatric and neurodevelopmental disorders, including autism spectrum disorder (ASD), attention deficit hyperactivity disorder (ADHD), obsessive-compulsive disorder (OCD), Tourette’s syndrome (TS), bipolar disorder, schizophrenia and mood disorders. The interaction between the genetic pathways governing the inhibitory/excitatory balance, neuromodulation, synaptic plasticity and immune interactions provides a complete framework for understanding the neurogenetic basis of sensory hypersensitivity in psychiatric conditions. Genetic alterations affecting the balance between excitatory (glutamatergic) and inhibitory (GABAergic) neurotransmission appear as central mechanisms underlying sensory hypersensitivity. Variants of the genes regulating the neuromodulation of serotonin and dopamine further influence sensory processing and emotional regulation. These pathways modulate the excitability of sensory neurons and synaptic function, contributing to an alteration of central sensory integration. High estimates of heritability and a genetic architecture shared with traits such as alexithymia highlight the convergence of sensory processing and the genetics of emotional regulation. In addition, neuro-immune signaling pathways interact with synaptic mechanisms to modulate the function and sensitivity of sensory neurons.

**Conclusions:**

Genetic research reveals a multifaceted molecular architecture leading to sensory hypersensitivity, highlighting the need for integrative approaches to unravel its role in psychiatric disorders and promote precision therapies.

**Disclosure of Interest:**

None Declared

